# Comparative Evaluation of Efficacy of Three Disinfectants on Polyvinyl Siloxane Impression Material: An In Vitro Study

**DOI:** 10.7759/cureus.113951

**Published:** 2026-08-04

**Authors:** Soham P Kothari, Shivsagar Tewary, Pronob Sanyal, Karuna Pawashe, Aaditee Vande, Harshada Ghadage

**Affiliations:** 1 Department of Prosthodontics, School of Dental Sciences, Krishna Vishwa Vidyapeeth Deemed to be University, Karad, IND

**Keywords:** cross-infection control, dimensional stability, glutaraldehyde, hypochlorous acid, impression disinfection, ozone, polyvinyl siloxane, streptococcus mutans

## Abstract

Background

Dental impressions represent a potential source of cross-contamination between clinical and laboratory settings. While disinfection protocols are essential for infection control, selecting an agent with reliable antimicrobial efficacy remains a key clinical consideration. This study aimed to compare the antimicrobial efficacy of three disinfection methods: 2% glutaraldehyde, Sterilox (hypochlorous acid-based), and gaseous ozone on polyvinyl siloxane (PVS) impression material.

Aim

The study aimed to determine and compare the antimicrobial efficacy of three disinfectants against *Streptococcus mutans* on PVS impressions.

Methods

Eighty disc-shaped PVS specimens were fabricated using a standardized stainless-steel mold and randomly allocated into four groups (n=20 each): control (no disinfection), 2% glutaraldehyde immersion (10 minutes), Sterilox immersion (10 minutes), and gaseous ozone exposure (30 minutes). Specimens were inoculated with *S. mutans*, and colony-forming units (CFUs) were quantified before and after disinfection. Statistical analysis employed one-way analysis of variance with post-hoc comparisons (α=0.05).

Results

All disinfectants produced statistically significant reductions in microbial load compared with controls (p<0.001). Glutaraldehyde demonstrated the highest CFU reduction (75.50±4.97), followed by Sterilox (69.20±3.05) and ozone (63.30±5.25). Intergroup differences were statistically significant (p<0.001), indicating variation in antimicrobial effectiveness among the tested agents.

Conclusions

Glutaraldehyde demonstrated superior antimicrobial efficacy. Sterilox offers a safer alternative with effective disinfection, while gaseous ozone shows promise as a residue-free modality but requires strict protocol standardization for consistent results.

## Introduction

Prosthodontic rehabilitation relies fundamentally on precise impression recording, which forms the basis for fabricating indirect restorations and prostheses. The Glossary of Prosthodontic Terms defines an impression as a negative replica of oral structures that, when cast, produces a positive working model [1].

Polyvinyl siloxane (PVS), an addition silicone elastomer, is widely used due to its favorable physical properties. Addition silicone impression materials are recognized for excellent dimensional stability and elastic recovery. Unlike condensation silicones, it polymerizes without releasing volatile byproducts [2,3]. Its superior elastic recovery allows accurate reproduction of fine details even after removal from undercut areas [4]. Contemporary formulations also include surfactants to improve hydrophilicity, enhancing wettability and reducing surface defects in casts. Surface detail reproduction remains an essential requirement for the clinical success of PVS impression materials.

However, infection control remains a critical concern. Impressions are routinely contaminated with saliva, blood, and oral microorganisms, making them potential sources of cross-contamination between clinicians and laboratory personnel. Studies have shown that simple rinsing is insufficient to eliminate microbial load, necessitating effective disinfection protocols capable of achieving significant microbial reduction or elimination [5,6]. Several simplified chairside disinfection protocols have been proposed for the addition of silicone impressions to improve routine clinical compliance. The efficacy of a disinfectant depends on its ability to act against a broad spectrum of microorganisms, including bacteria, fungi, and viruses, while maintaining consistent performance under clinical conditions.

Guidelines from organizations such as the Centers for Disease Control and Prevention, American Dental Association, and World Health Organization recommend immediate disinfection of all impressions [2]. However, the choice of disinfectant must balance antimicrobial potency with safety and clinical practicality [7]. Conventional agents such as glutaraldehyde and sodium hypochlorite are well-established for their high-level disinfection capabilities, acting through protein denaturation and oxidative mechanisms that rapidly inactivate microbial cells [4]. Newer alternatives such as Sterilox (hypochlorous acid-based) and gaseous ozone offer promising antimicrobial activity through oxidative disruption of microbial cell structures, with potential advantages in biocompatibility and ease of use [8,9].

Given the limited and inconsistent literature, this in vitro study aimed to compare the antimicrobial efficacy of 2% glutaraldehyde, Sterilox, and gaseous ozone against *Streptococcus mutans* on PVS, addressing the critical aspect of infection control in prosthodontic practice [10].

## Materials and methods

Study design and ethical considerations

This in vitro comparative study was conducted in the Department of Prosthodontics and Crown and Bridge at Krishna Vishwa Vidyapeeth (Deemed to be University), Karad, Maharashtra, India. The protocol received institutional approval (Protocol No. 025/2024-2025) and ethical clearance from the Institutional Ethics Committee (Ref. No. KVV/IEC/10/2024, dated August 3, 2024).

Sample size determination and group allocation

A total of 80 PVS specimens were fabricated and randomly distributed into four experimental groups (n=20 per group): (1) group 1 (control): no disinfection intervention, (2) group 2 (glutaraldehyde): immersion in 2% glutaraldehyde solution for 10 minutes, (3) group 3 (Sterilox): immersion in Sterilox (hypochlorous acid-based) solution for 10 minutes, and (4) group 4 (ozone): exposure to direct gaseous ozone flow for 30 minutes.

Specimen fabrication

**Figure 1:**
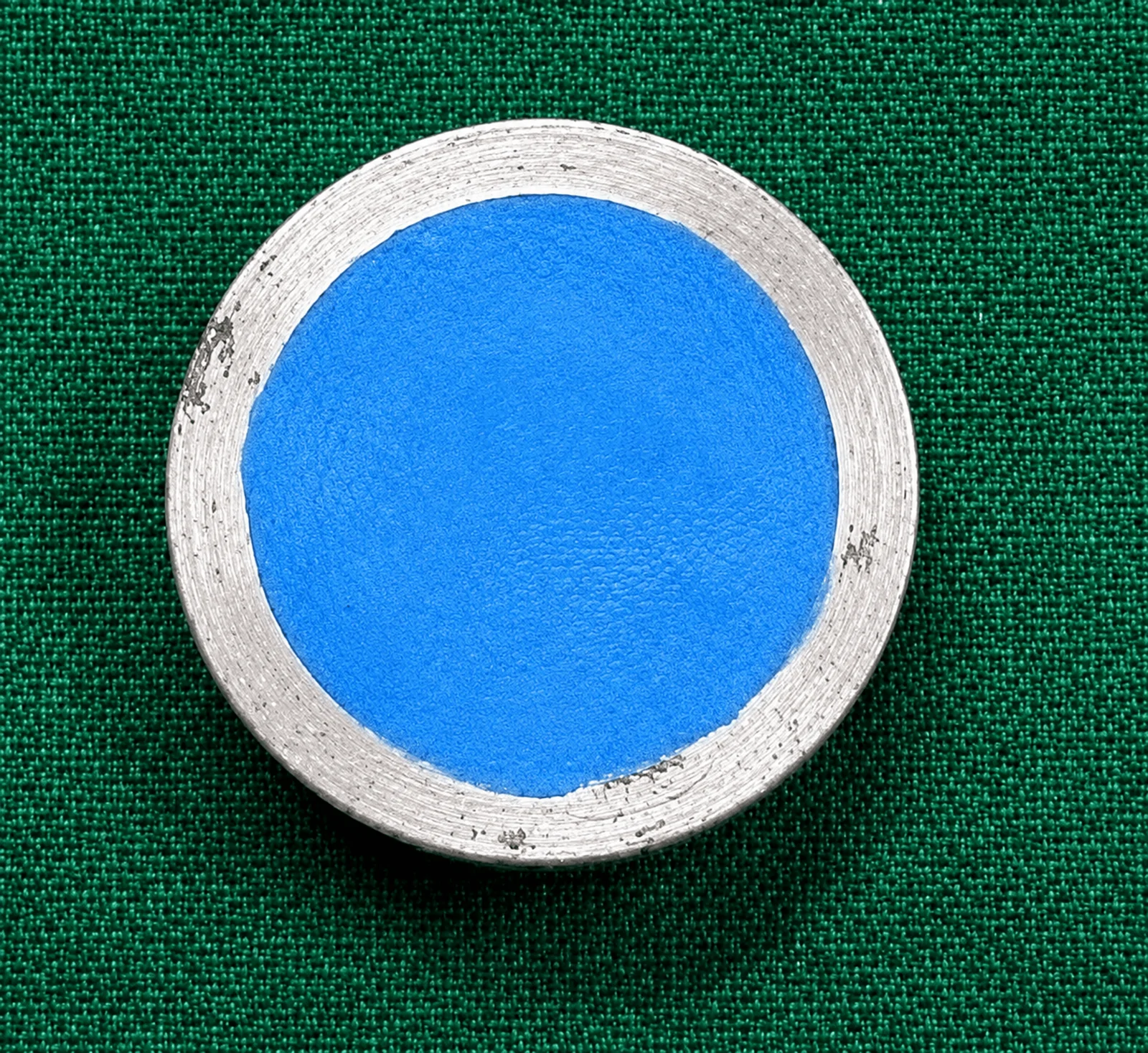
Fabrication of specimens

A standardized stainless-steel mold (4 mm depth, 19 mm diameter) was used to fabricate uniform disc-shaped PVS specimens (Figure 1). The material was mixed as per manufacturer instructions and allowed to set under controlled conditions, and specimens with defects were excluded after inspection.

Microbial inoculation and baseline assessment

**Figure 2:**
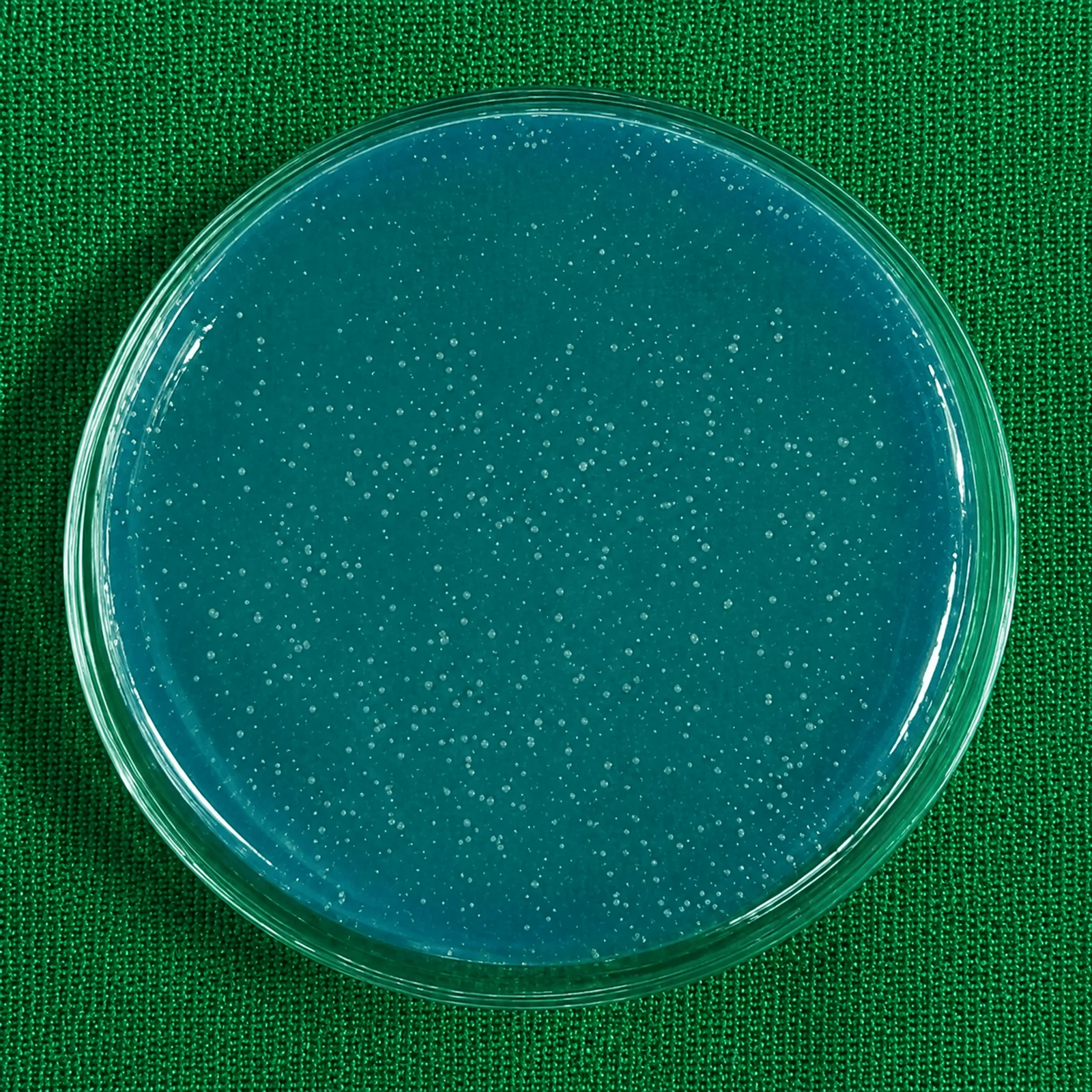
Checking microbial count before disinfection with gaseous ozone

**Figure 3:**
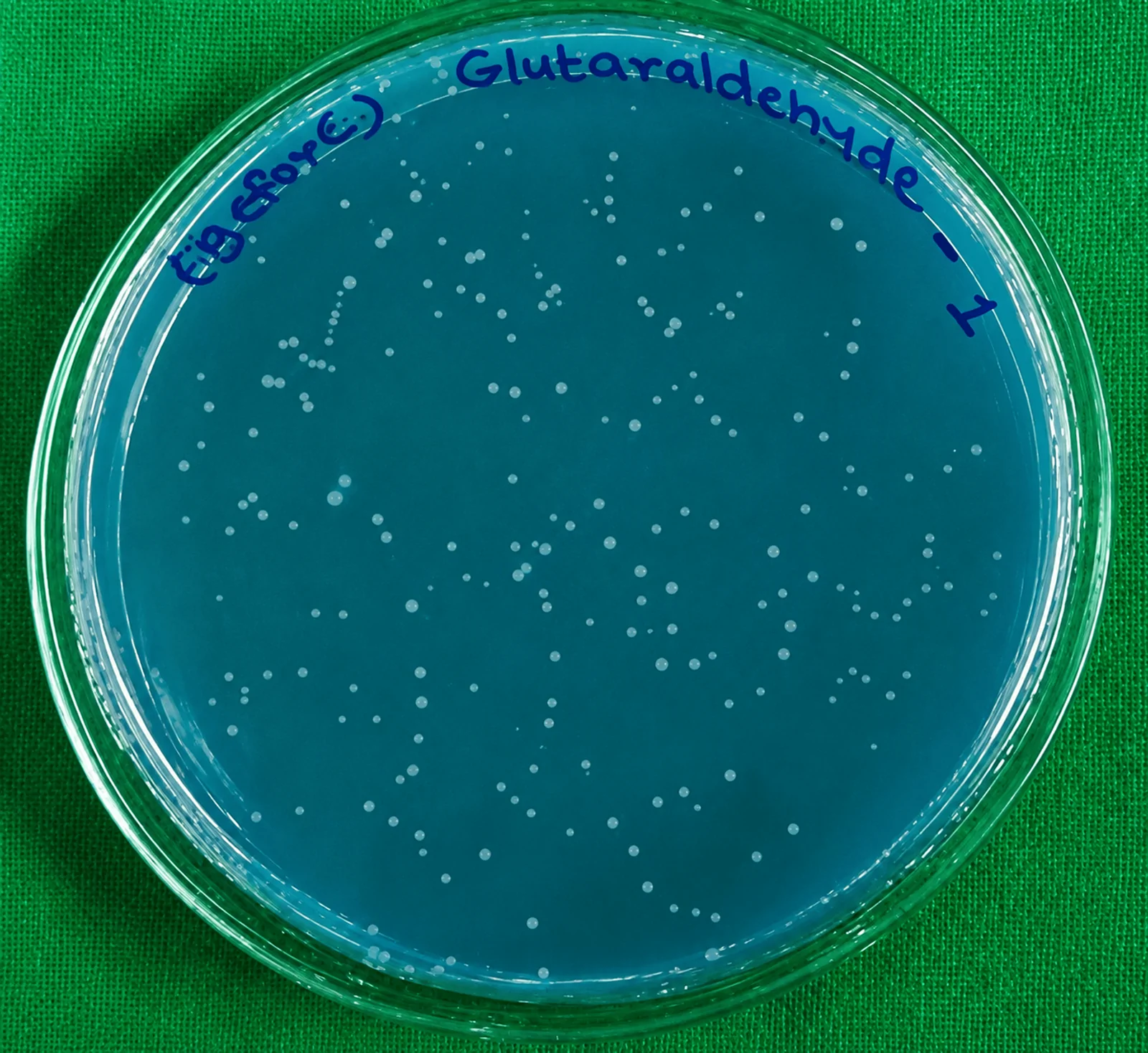
Checking microbial count before disinfection with 2% glutaraldehyde

**Figure 4:**
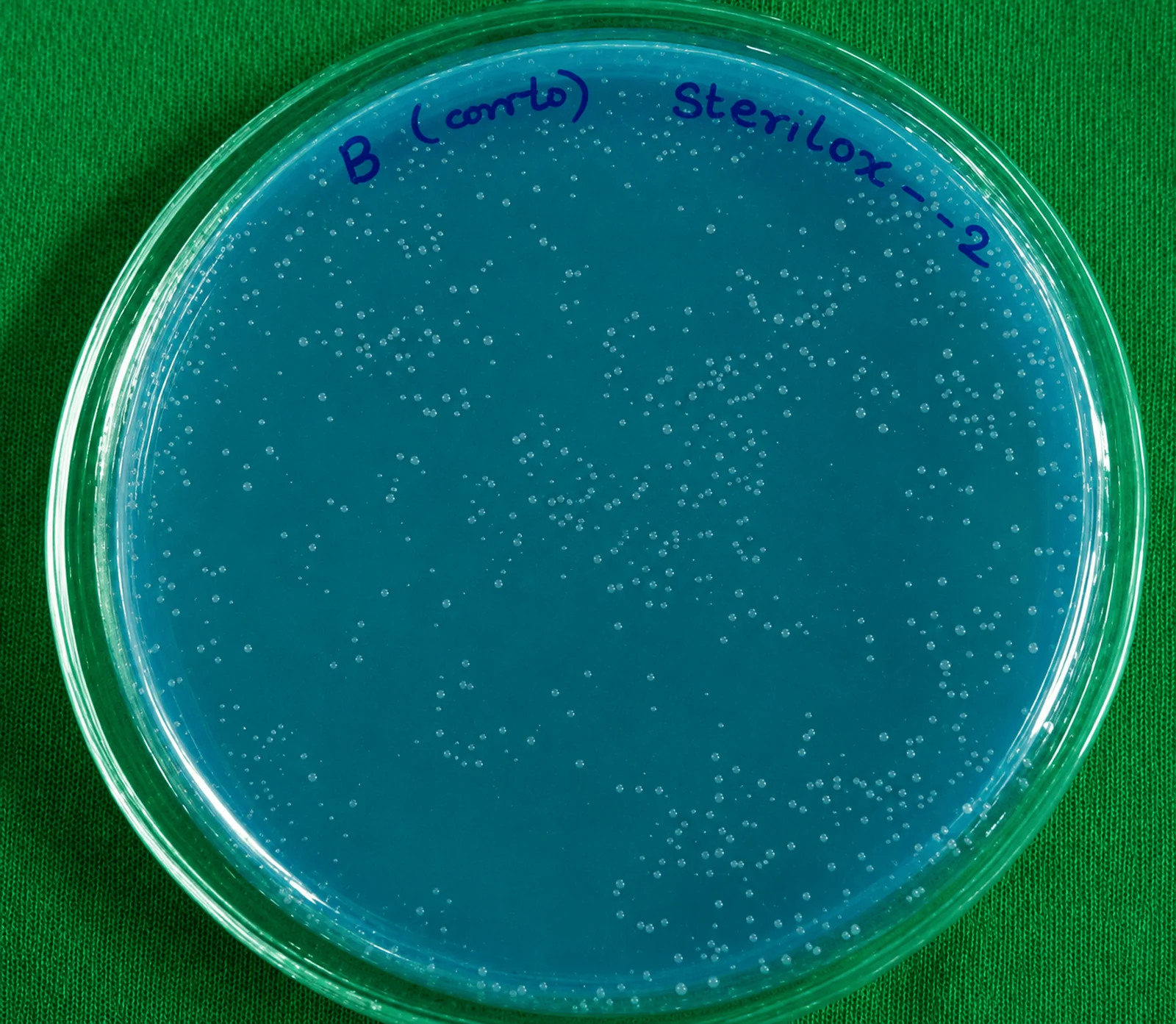
Checking microbial count before disinfection with Sterilox

All specimens were inoculated with a standardized *S. mutans* suspension in Brain Heart Infusion broth and incubated at 37°C for 24 hours. Baseline microbial load was then assessed using the colony-forming unit (CFU) method by swabbing specimens, culturing on Mueller-Hinton agar, and counting colonies after 24-hour incubation with a digital colony counter (Figures 2-4).

Disinfection protocol

**Figure 5:**
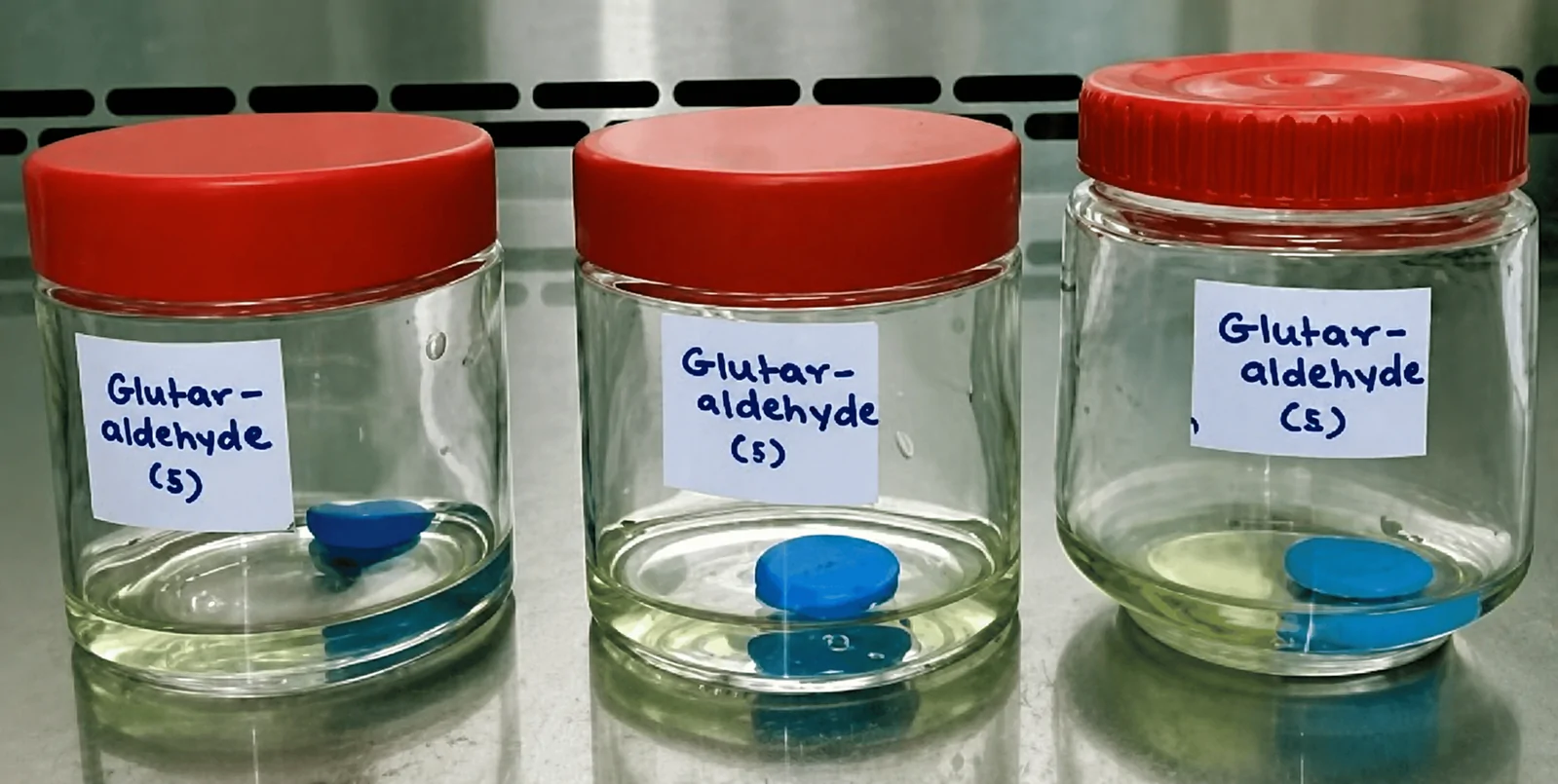
Disinfection of specimens with 2% glutaraldehyde disinfectant

**Figure 6:**
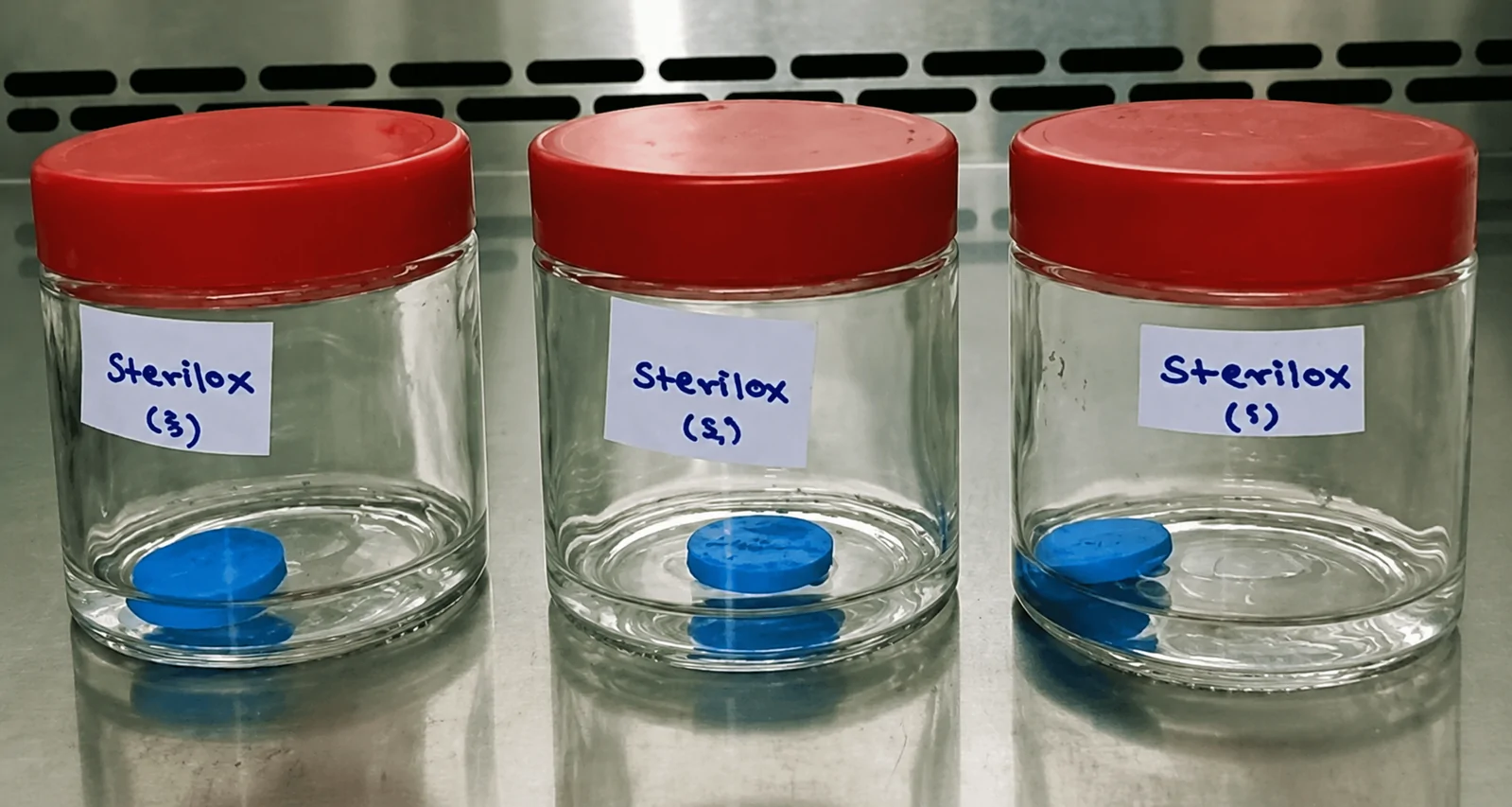
Disinfection of specimens with Sterilox disinfectant

**Figure 7:**
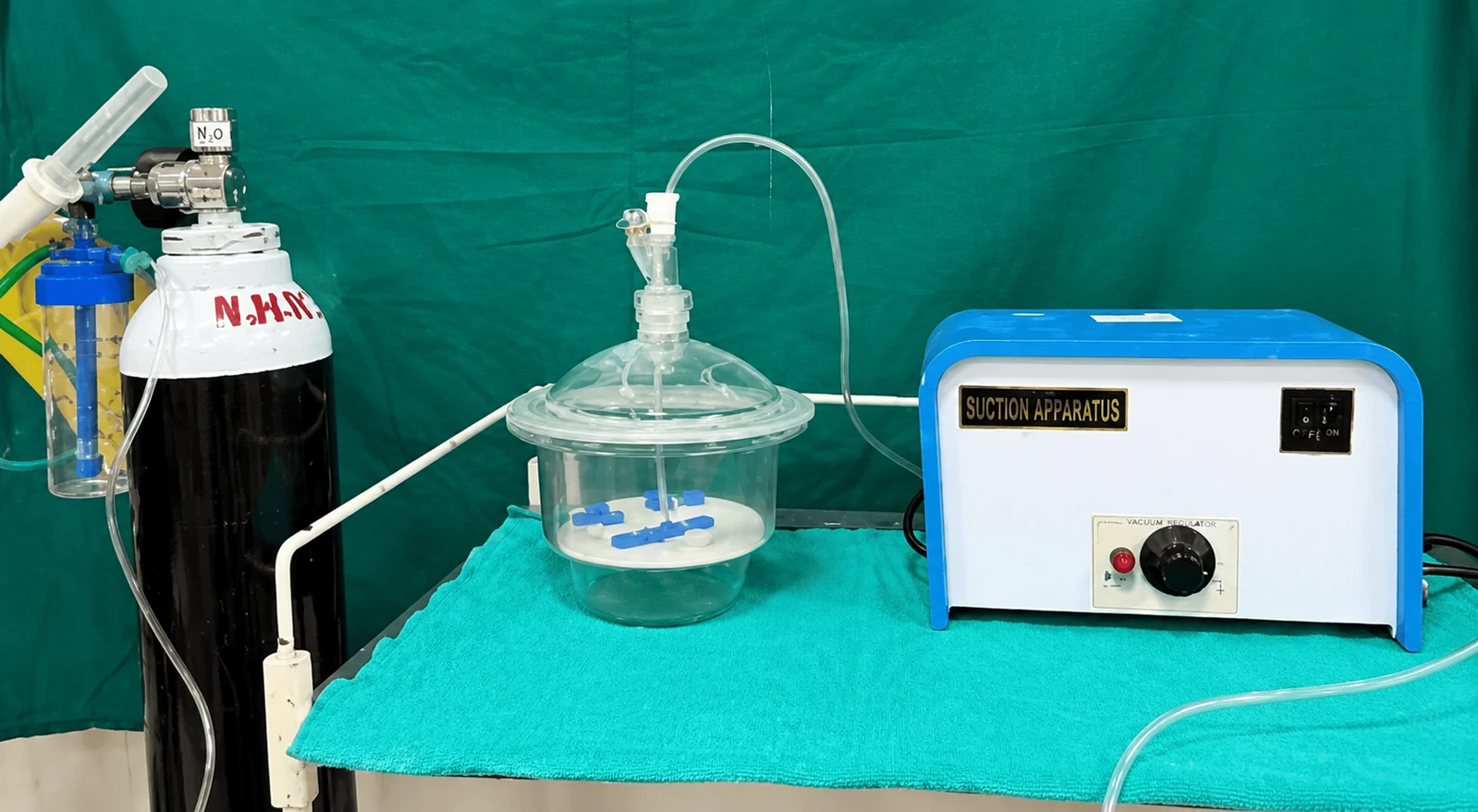
Disinfection of specimens with gaseous ozone disinfectant

Post-baseline assessment, specimens underwent disinfection according to their allocated group: (1) control group: specimens received no disinfection treatment, (2) glutaraldehyde group: complete immersion in 2% glutaraldehyde solution for 10 minutes, (3) Sterilox group: complete immersion in Sterilox solution for 10 minutes, (4) ozone group: placement within a sealed glass desiccator chamber with direct gaseous ozone flow from the generator for 30 minutes (Figures 5-7).

Following disinfection, specimens in immersion groups were rinsed with sterile distilled water and air-dried under aseptic conditions within the laminar airflow chamber.

Post-disinfection microbial evaluation

**Figure 8:**
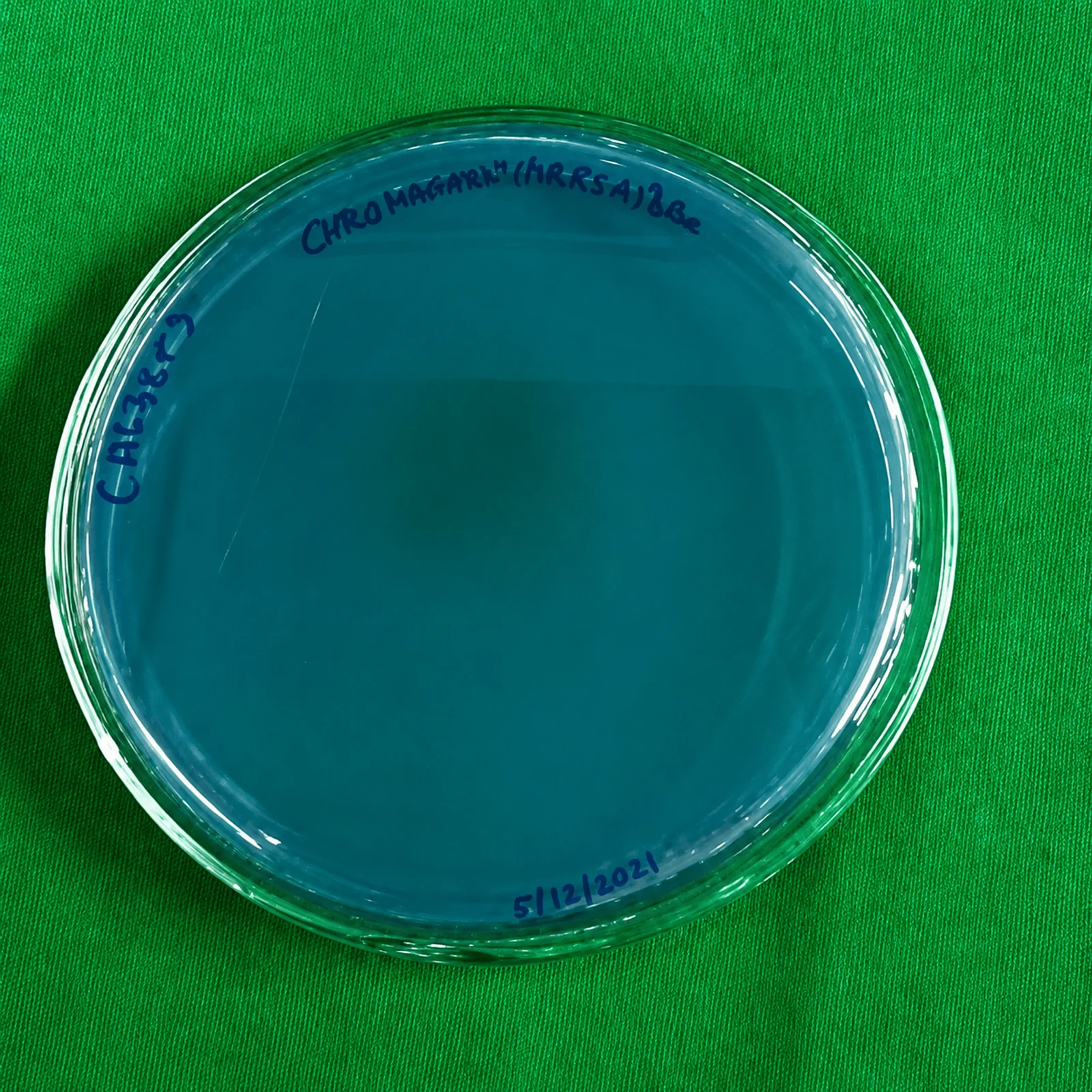
Checking microbial count after disinfection with 2% glutaraldehyde

**Figure 9:**
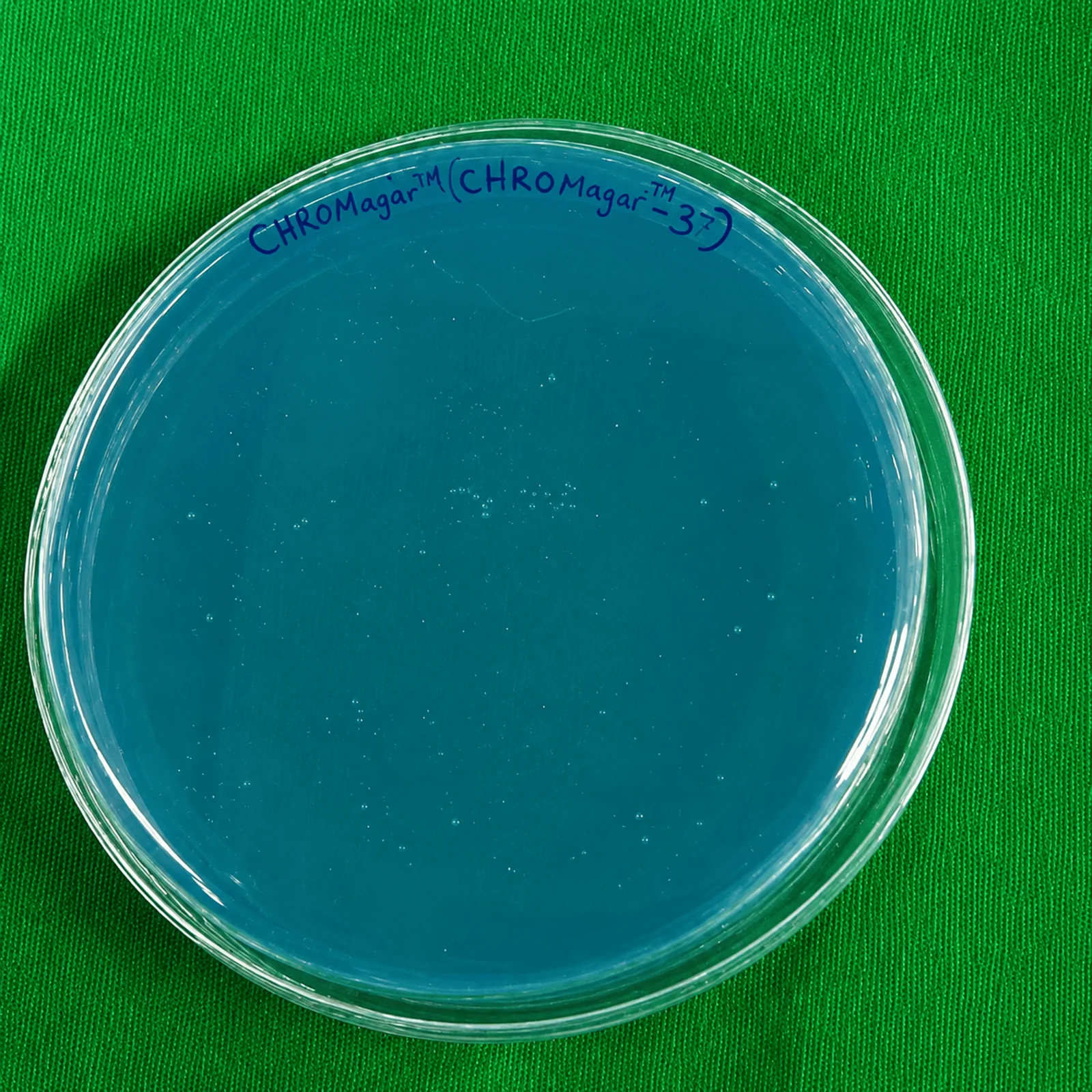
Checking microbial count after disinfection with Sterilox

**Figure 10:**
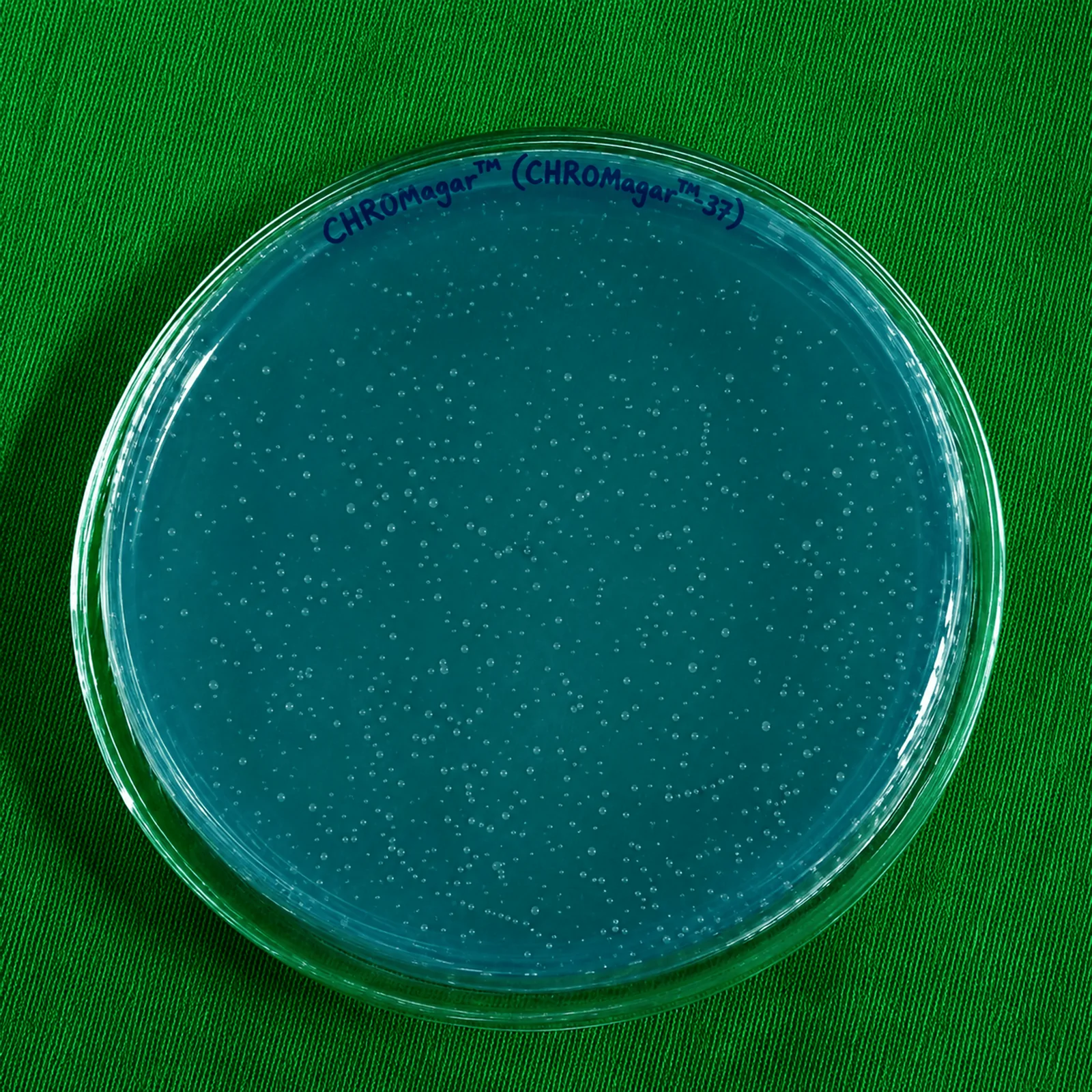
Checking microbial count after disinfection with gaseous ozone

Antimicrobial efficacy assessment was repeated using identical CFU methodology. Post-disinfection colony counts were recorded and compared with baseline values to calculate microbial reduction for each disinfectant group (Figures 8-10).

Statistical analysis

Data were analyzed using IBM SPSS Statistics version 25.0 (IBM Corp., Armonk, NY). Descriptive statistics (mean, standard deviation) were calculated, and intergroup comparisons were performed using one-way analysis of variance (ANOVA) with Tukey’s post-hoc and independent t-tests. Statistical significance was set at α=0.05 (p>0.05: not significant; p≤0.05: significant; p<0.001: highly significant).

## Results

Microbial reduction by different disinfection methods

Descriptive statistics for microbial reduction across disinfectant groups are presented in Table 1. Glutaraldehyde achieved the highest mean CFU reduction (75.50±4.97), demonstrating superior antimicrobial performance with relatively consistent results across specimens, as indicated by the low standard deviation. Sterilox produced a moderate reduction (69.20±3.05), while gaseous ozone exhibited the lowest reduction among active disinfectants (63.30±5.25).

**Table 1:** Comparison of disinfection methods on Streptococcus mutans CFU, colony-forming unit.

Disinfectant	Mean reduction (CFU/mL)	Standard deviation
Glutaraldehyde	75.50	4.97
Sterilox	69.20	3.05
Ozone	63.30	5.25

Pairwise comparison of antimicrobial efficacy

Pairwise statistical comparisons using independent t-tests revealed significant differences between all disinfectant combinations (Table 2). The comparison between glutaraldehyde and ozone yielded the highest t-statistic (5.34, p<0.0001), indicating the most pronounced efficacy difference.

**Table 2:** Pairwise comparison of disinfectants (t-test results)

Comparison	t-statistic	p-value	Significance
Glutaraldehyde vs Sterilox	3.42	0.0031	Significant
Glutaraldehyde vs ozone	5.34	0.0000	Significant
Sterilox vs ozone	3.07	0.0066	Significant

Overall comparison among disinfectant groups

One-way ANOVA confirmed highly significant overall differences among the three disinfectant groups (F=18.13, p<0.0001), as shown in Table 3.

**Table 3:** One-way ANOVA result for antimicrobial efficacy ANOVA, analysis of variance.

Test	F-statistic	p-value
One-way ANOVA	18.13	0.0000

## Discussion

The present investigation addressed a key clinical challenge in prosthodontics: achieving effective impression disinfection without compromising clinical performance. The evaluation of antimicrobial efficacy reflects clinical reality, where adequate reduction of microbial load is essential for preventing cross-contamination and ensuring biosafety during prosthesis fabrication.

Glutaraldehyde demonstrated the highest antimicrobial efficacy, consistent with previous studies reporting near-complete bacterial elimination with 2% glutaraldehyde [10,11]. Its mechanism involves protein coagulation and alkylation of microbial amino groups, leading to irreversible enzymatic inactivation [5]. This dual mode of action enables glutaraldehyde to disrupt both structural and metabolic functions of microorganisms, making it highly effective against a broad spectrum of bacteria, fungi, and viruses. Studies by Al-Jabrah [12] further confirmed its ability to achieve high-level disinfection. Clinically, this is significant, as impressions are known vectors for cross-contamination between clinics and laboratories [13,14].

Gaseous ozone exhibited the lowest antimicrobial efficacy among the tested agents, though still significantly better than controls. Its mechanism involves oxidation of microbial cell structures, including lipids, proteins, and nucleic acids [15,16]. Ozone has demonstrated antimicrobial activity against a broad spectrum of oral microorganisms in previous microbiological studies [15,16]. Ozone can cause rapid cell lysis and damage to genetic material; however, its effectiveness is influenced by factors such as concentration, exposure time, and uniformity of gas distribution [16]. Variability in gaseous diffusion and exposure may result in inconsistent microbial reduction, particularly in areas with complex surface morphology. This technique's sensitivity suggests that strict protocol control is essential for achieving consistent antimicrobial outcomes with ozone. Previous studies evaluating ozone gas on PVS impressions have similarly demonstrated significant antibacterial activity with variable efficacy outcomes [17]. Recent studies comparing ozone gas and glutaraldehyde on PVS materials have reported findings consistent with the present investigation.

Sterilox showed substantial, though comparatively lower, antimicrobial activity. Its hypochlorous acid component exerts oxidative effects on microbial cell walls, membrane lipids, and intracellular components [17]. In addition to membrane disruption, hypochlorous acid interferes with essential enzymatic pathways, leading to rapid microbial death. While slightly less potent than glutaraldehyde, Sterilox remains clinically effective and offers improved safety. McDonnell and Russell [3] highlighted concerns associated with aldehyde exposure, including mucosal and respiratory irritation, supporting Sterilox as a safer alternative in routine practice.

Disinfectant selection should consider antimicrobial efficacy, operator safety, and clinical practicality [7]. Previous investigations have also demonstrated that disinfectants may influence the dimensional accuracy of elastomeric impression materials [13]. Glutaraldehyde offers superior microbial elimination but requires caution due to occupational hazards [5,11]. Sterilox provides a balanced profile with effective disinfection and improved safety, making it suitable for routine use. Ozone presents a residue-free alternative, though its effectiveness depends on strict protocol standardization.

The findings align with previous studies demonstrating the superior antimicrobial efficacy of glutaraldehyde [14] and the effectiveness of hypochlorous acid systems [5,16]. The protocol sensitivity of ozone observed here is consistent with reports emphasizing controlled exposure conditions [11,17]. Variations among studies may result from differences in disinfectant concentration, exposure duration, and testing methodologies. The use of *S. mutans* enhances clinical relevance, though real-world contamination is polymicrobial [14].

A major strength of this study is its dual evaluation design, simultaneously assessing antimicrobial efficacy. Standardized methodology, including specimen preparation, contamination, and measurement, enhanced reliability and reproducibility. The comparison of three disinfectants with distinct mechanisms further strengthens the findings [11].

Limitations include the in vitro design, which cannot fully replicate oral conditions such as saliva, blood, and mixed microbial flora. The use of a single bacterial strain does not reflect polymicrobial biofilms observed clinically [14]. Although cost analysis was not an objective of the present study, 2% glutaraldehyde is generally considered the most cost-effective disinfectant because of its low cost and easy availability. In contrast, Sterilox and gaseous ozone require specialized generation units, increasing the initial investment, with ozone systems typically incurring the highest equipment and maintenance costs among the three methods.

Alternative sterilization techniques, including heat sterilization, have also been investigated for PVS materials [15]. Future studies should incorporate multi-species biofilm models. Advanced analyses such as scanning electron microscopy could provide deeper insight into surface interactions. Clinical trials evaluating prosthesis fit and the effect of disinfectant exposure on the wettability characteristics of impression materials would further enhance the clinical applicability of these findings by assessing their potential influence on cast quality [7,16].

## Conclusions

Within the limitations of this in vitro study, all three disinfection methods demonstrated the ability to reduce microbial contamination of impression materials. Among them, glutaraldehyde exhibited the highest antimicrobial efficacy while maintaining minimal dimensional distortion, indicating its superior overall performance. Sterilox provided effective microbial reduction with the added advantage of a safer handling profile, supporting its suitability for routine clinical application. Gaseous ozone also achieved significant microbial reduction without leaving chemical residues; however, its technique-sensitive nature necessitates strict protocol standardization to ensure consistent outcomes.

The findings of this study highlight that the selection of an impression disinfection method should be based not only on antimicrobial effectiveness but also on its impact on dimensional accuracy, material compatibility, operator safety, and clinical feasibility. Further clinical investigations are warranted to validate these findings under in vivo conditions and to establish standardized disinfection protocols that optimize both infection control and impression accuracy.
